# Connectivity motifs of inhibitory neurons in the mouse Auditory Cortex

**DOI:** 10.1038/s41598-017-16904-2

**Published:** 2017-12-05

**Authors:** Hysell V. Oviedo

**Affiliations:** The City College of New York, Biology Department, New York, NY USA

## Abstract

Connectivity determines the function of neural circuits and it is the gateway to behavioral output. The emergent properties of the Auditory Cortex (ACx) have been difficult to unravel partly due to our assumption that it is organized similarly to other sensory areas. But detailed investigations of its functional connectivity have begun to reveal significant differences from other cortical areas that perform different functions. Using Laser Scanning Photostimulation we previously discovered unique circuit features in the ACx. Specifically, we found that the functional asymmetry of the ACx (tonotopy and isofrequency axes) is reflected in the local circuitry of excitatory inputs to Layer 3 pyramidal neurons. In the present study we extend the functional wiring diagram of the ACx with an investigation of the connectivity patterns of inhibitory subclasses. We compared excitatory input to parvalbumin (PV) and somatostatin (SOM)-expressing interneurons and found distinct circuit-motifs between and within these subpopulations. Moreover, these connectivity motifs emerged as intrinsic differences between the left and right ACx. Our results support a functional circuit based approach to understand the role of inhibitory neurons in auditory processing.

## Introduction

The cerebral cortex guides behavior by extracting salient sensory signals. These functions are executed by cortical modules that share many features (laminar structure, cell types, connectivity motifs) but also contain significant specializations. By relating detailed connectivity information to sensory coding *in vivo*, we are beginning to identify some of the specialized circuit motifs embedded in stereotyped modules that enable different cortical areas to perform distinct tasks. In a previous study we compared the connectivity between orthogonal functional axes in the Auditory Cortex (tonotopy and isofrequency) and found unique circuit-motifs in excitatory networks along the tonotopic axis that were absent along the isofrequency axis. These findings suggest that auditory cortical microcircuitry is specialized to the unique one-dimensional representation of frequency in the Auditory Cortex (ACx)^1^. Other studies have also challenged the idea that cortical circuits are homogeneous processing modules with identical flow of information^2–4^. Compared to the visual and sensorimotor cortices, the connectivity in the Auditory Cortex is not as well understood. The full connectivity of most auditory circuits and their role in sound perception remain unknown. Here, we continue to fill this gap by mapping the functional synaptic connectivity of two genetically identified inhibitory cell classes.

Inhibitory interneurons are the most diverse cell-type in cortex whose task is to control the flow of activity within local circuits. Interneurons can be classified according to molecular, morphological, and biophysical properties, though with many ambiguities^5^. Nevertheless, three genetically defined sub-classes have dominated recent studies of inhibitory function thanks to readily available cre-driver transgenic mouse lines^6^. Here we compare the connectivity of two sub-classes: parvalbumin (PV) and somatostatin-positive (SOM) inhibitory neurons. In addition to being molecularly and morphologically distinct, these subclasses also employ different mechanisms to control neural activity. Using cre-driver lines and optogenetic tools, researchers have begun to dissect the function of these cell classes in neural computations. Some of their proposed functions include shaping receptive fields^7^, gain control, and surround suppression^8,9^. However, in the ACx the laminar and cell-type dependent tuning properties of excitation and inhibition remain under debate^7,10^. The lack of knowledge of the functional connectivity of inhibitory neurons in the ACx has hindered our ability to build detailed models or reject existing models of their role in auditory function. The diverse circuit-motifs of inhibitory neurons in other cortical areas supports the need for this information in the ACx^11,12^. Although molecular classification schemes are helpful, circuit function is a more useful way to classify neurons^13^. In support of this proposal, we found distinct circuit-motifs in the connectivity of PV and a sub-population of SOM inhibitory neurons in the left and right ACx. These findings underscore the need for connectivity-based and region-specific insight of inhibitory microcircuits in cortex.

## Methods

**Figure 1:**
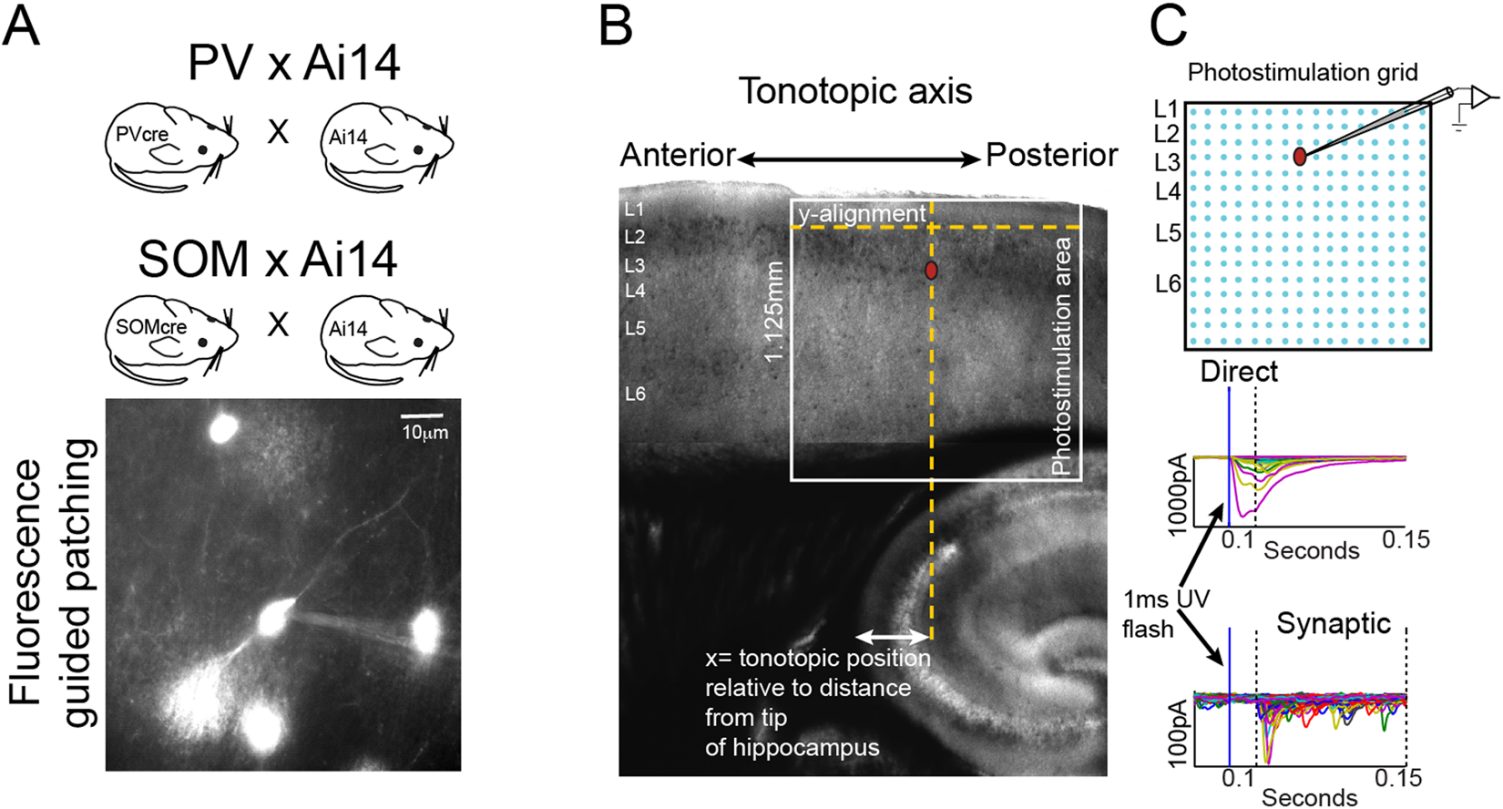
Cell-type specific mapping using Laser Scanning Photostimulation (LSPS). (**A**) Ai14 reporter mice are crossed with PV and SOM inhibitory cre-driver lines (top) to perform cell-type specific, fluorescence-guided patching (bottom). (**B**) 4x picture of a tonotopic slice visualized using infrared gradient contrast optics showing the LSPS stimulation grid and anatomical landmarks used for consistent mapping. (**C**) Using patch-clamp recordings in neuronal classes of interest, we map the strength and organization of their presynaptic inputs. Photostimulation grid (top) shows the 16 × 16 points of uncaging. Grids are centered on the soma of the cell mapped. (Bottom) examples of direct responses (i.e. those evoked by the direct activation of the neuron under study) and examples of synaptic responses (i.e. EPSCs elicited by triggering action potentials in neurons presynaptic to the neuron under study). The vertical lines through the traces mark the time window to detect direct responses (first 2 vertical lines) and synaptic events (second and third vertical lines).

The care and handling of the animals, experiments and methods were done in accordance with a protocol (#10-07-4) approved by the Institutional Animal Care and Use Committee (IACUC) of Cold Spring Harbor Laboratory. To visualize inhibitory cell types, we bred PV and SOM cre-lines with the Ai14-tdTomato reporter mouse (Jackson labs), and combined differential interference contrast microscopy with fluorescence to guide whole-cell recordings (Fig. 1A). Male mice aged > 21 days postnatal were anesthetized, decapitated, and their brains transferred to a chilled cutting solution composed of (in mM): 110 choline chloride, 25 NaHCO_3_, 25 D-glucose, 11.6 sodium ascorbate, 7 MgCl_2_, 3.1 sodium pyruvate, 2.5 KCl, 1.25 NaH_2_PO_4_, and 0.5 CaCl_2_. We made 300 μm thick horizontal slices cut at a 15-degree angle between the blade and the medial-lateral axis of the brain^14^. This preparation preserves tonotopy: the anterior-posterior representation of frequencies in ACx. Slices were then transferred to artificial cerebrospinal fluid (ACSF) containing (in mM): 127 NaCl, 25 NaHCO_3_, 25 D-glucose, 2.5 KCl, 1 MgCl_2_, 2 CaCl_2_, and 1.25 NaH_2_PO_4_, aerated with 95% O_2_ 5% CO_2_. The slices were incubated at 34° for 20–30 minutes and then kept at room temperature during the experiments. The ACx lacks clear anatomical landmarks to align the stimulus grid for each cell, which is essential to establish consistent tonotopic position across animals. To overcome this challenge, we developed a systematic coordinate system. We center the x-axis of the photostimulation grid on the soma of the neuron recorded and align the y-axis with the second row of the grid on the L1/2 border, the clearest laminar boundary in our slices. For each cell, we measure spatial coordinates: distance from the soma to the pial surface, L1/2 border, and the horizontal distance to the anterior tip of the hippocampus (where the fimbria exits). The latter measurement is used to assign each cell a position along the anterior-posterior tonotopic axis (Fig. 1B).

### *In vitro* physiology and LSPS

We performed fluorescence-guided patching of inhibitory neurons 45–80 μm below the surface of the slice visualized using infrared gradient contrast optics with electrodes (6–7 MΩ) containing the following intracellular solution (in mM) 128 K-methylsulfate, 4 MgCl_2_, 10 HEPES, 1 EGTA, 4 Na_2_ATP, 0.4 Na_2_GTP, and 10 Na-phosphocreatine; pH 7.25, 300 mOsm). The recording artificial cerebrospinal fluid contained (in mM) 0.37 nitroindolinyl (NI)-caged glutamate (Tocris), 0.005 CPP (Tocris), 4 CaCl_2_ and 4 MgCl_2_. The high divalent concentration and CPP (NMDA channel blocker) prevent reverberant excitatory activity due to photostimulation. Voltage-clamp recordings were made using a Multiclamp 700 A amplifier (Axons Instruments, Molecular Devices, Sunnyvale, California, USA). We photoreleased the caged glutamate compound with a 1-ms light stimulus consisting of 100 pulses from a pulsed UV laser (wavelength, 355 nm; repetition rate, 100 kHz, DPSS Lasers, Santa Clara, California USA). To map hotspots of presynaptic input we used a 16 × 16 stimulus grid with 75 μm spacing, resulting in a mapping region of 1.125 × 1.125 mm. Hotspots in the maps correspond to areas that connect monosynaptically to the recorded cells.

### LSPS analysis

Detailed analyses were published previously^1^. Briefly, the mean current amplitude of Excitatory Postsynaptic Currents (EPSCs) was calculated in the 50 ms epoch after the direct response time window (7.5 ms after UV stimulus; Fig. 1C). The values for each site stimulated are represented as pixels in a colormap. Black pixels represent direct hits on the cell recorded and are excluded from analysis. We recorded 2 to 4 maps for each cell to create an average input map, and these average maps were used for group averages and for all analyses. All data shown is ± S.E.M. unless otherwise noted.

### Asymmetry analysis

To quantify the spatial asymmetry of synaptic input arising from infragranular layers, we divided the average population input maps in half along the anterior-posterior axis. We called one half Anterior Input and the other Posterior Input. To compute the Asymmetry Index for infragranular laminar input, we only included data points within 50% of the highest input. The Asymmetry Index is given by:

Asymmetry Index = (Anterior Input – Posterior Input)/(Anterior Input + Posterior Input)^15^.

An asymmetry index of 0 would indicate synaptic input centered on the soma of the cell mapped, whereas values between 0 and ±1 would indicate spatially/tonotopically shifted synaptic input.

### Data availability

All raw data and basic analysis software will be made available upon request to the author.

## Results

We previously characterized the connectivity of Layer 2 and 3 pyramidal neurons and found distinct connectivity patterns between these populations of principal neurons in horizontal slices of the ACx that preserve tonotopy. Because we found out-of-column circuit-motifs unique to the ACx in excitatory populations, we now focused on mapping the connectivity of inhibitory neurons in L3 of the ACx. Furthermore, we explored the potential for hemispheric differences in connectivity given the hypothesized functional division of labor between the left and right ACx. To target genetically defined inhibitory subclasses, we used two well-established transgenic mouse lines that express cre-recombinase in parvalbumin-expressing (PV), and somatostatin-expressing (SOM) inhibitory neurons. Together with vasoactive intestinal peptide-expressing (VIP) cells, these markers account for 84% of all inhibitory neurons in cortex and are believed to capture largely non-overlapping, molecularly-defined populations^11,16^. To isolate excitatory input onto these interneuron subclasses, we voltage-clamped cells near the inhibitory reversal potential (−70 mV).

### Connectivity of PV interneurons in L3 of the left and right ACx

**Figure 2:**
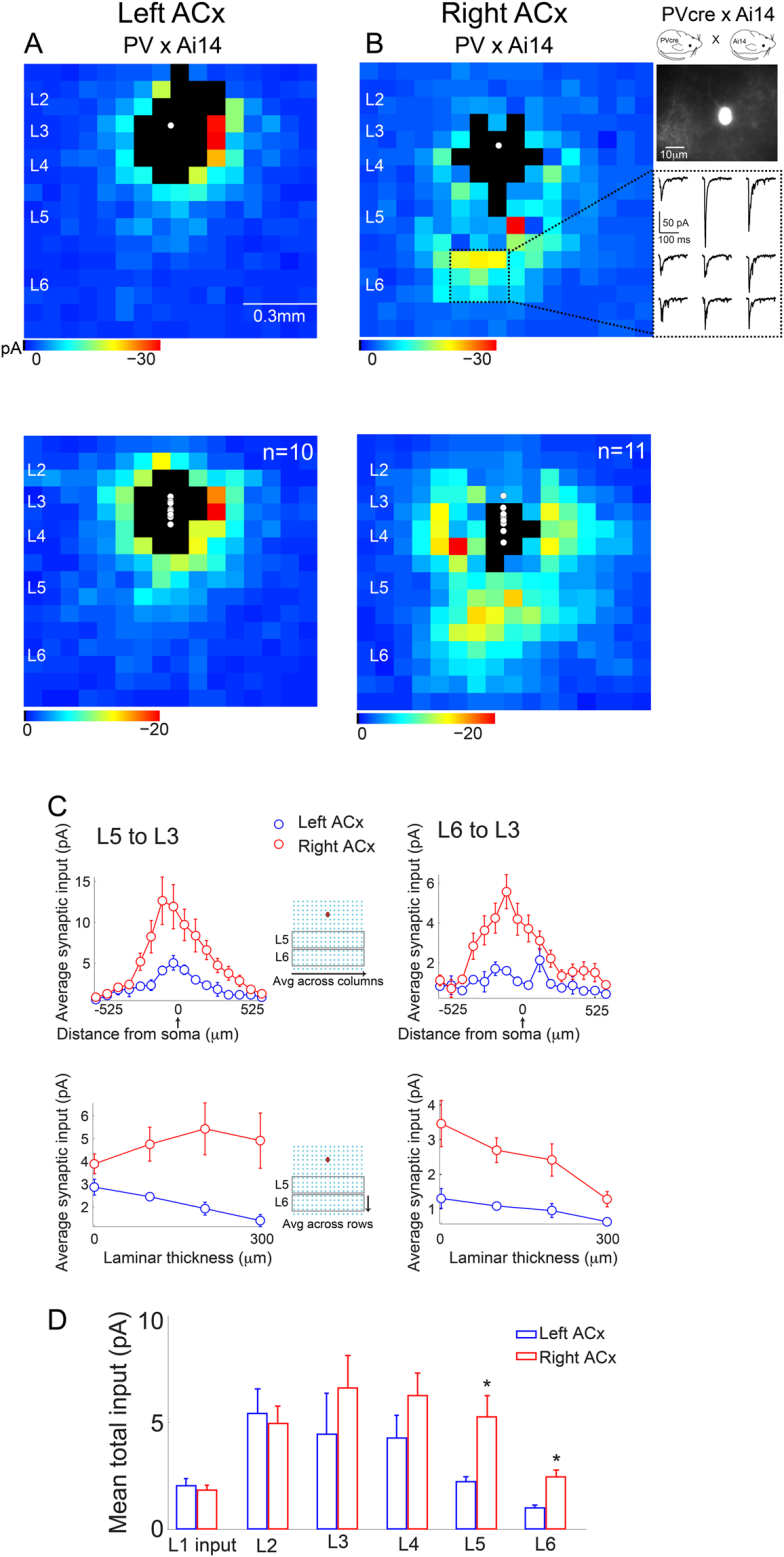
Connectivity of PV interneurons in L3 of the ACx. (**A**,**B** top) Representative single cell input maps of PV interneurons in L3 of the left and right ACx, respectively. Black pixels in maps represent responses from direct stimulation of the cells recorded and are excluded from analysis. Maps shown are of mean synaptic input averaged from 2 maps. Panels on the far right show a picture of the PV cell mapped in the right ACx and representative traces of L5/6 synaptic input. (**A**,**B** bottom) Population maps for the left ACx (n = 10 cells from 3 mice), and right ACx (n = 11 cells from 3 mice). (**C**, top) Left-Right ACx comparison of average synaptic input across the tonotopic axis (columnar average), and across infragranular layers (row average). (**D**) Left-Right ACx comparison of total synaptic input to L3 PV neurons from all layers. Input from L5 and L6 in the right ACx is significantly different between the hemispheres.

PV-expressing interneurons make up the largest population of inhibitory neurons. They tend to be fast-spiking, and target the cell body and axon hillock of pyramidal neurons. Although this subclass includes chandelier cells, PV-cre transgenic lines typically only capture basket cells. Furthermore, basket cells have been further sub-divided based on distinct patterns of axonal arborization^17^. A representative map of the average presynaptic input for a PV cell in L3 of the left ACx is shown in Fig. 2A (top). The map shows excitatory presynaptic input that largely arises from neighboring, recurrent connections and L4. Averaging a population of PV cells confirms this observation (Fig. 2A, bottom, n = 10 cells from 3 mice). This pattern of connectivity is consistent with what has been previously reported for PV interneurons in the visual cortex using LSPS^12^, and using pair-recordings in the ACx^18^.

To determine if a similar pattern of recurrent and L4 feedforward connectivity is conserved in the right ACx, we mapped a population of Layer 3 PV cells in horizontal slices. Unexpectedly, we found a significantly different circuit-motif in the right ACx. A representative input map shows that in addition to recurrent and feedforward input from L4, there is substantial infragranular input arising from L5/6 (Fig. 2B, top). This observation was consistent across the population of cells mapped (Fig. 2B, bottom, n = 11 cells from 3 mice). Quantitative analyses of input along the tonotopic axis and infragranular layers between the left and right ACx showed an increase in excitatory synaptic input to L3 PV interneurons beginning in middle portions of L5 (*p* = *0*.*03*, ranksum n = 21 from 6 mice; Fig. 2C, left) that became even more significant in L6 of the right ACx (*p* = *0*.*0042*, ranksum n = 21 from 6 mice; Fig. 2C, right). Input from other layers was comparable between the left and right ACx (input from L1: *p* = *0*.*78*, L2: *p* = *0*.*85*, L3: *p* = *0*.*45*, L4: *p* = *0*.*32*; ranksum n = 21 from 6 mice; Fig. 2D). The spatial/tonotopic organization of the infragranular input also appeared to have a spatial bias (Fig. 2C); therefore, we calculated an asymmetry index for the input arising from these pathways (see Methods). The asymmetry index revealed that infragranular synaptic input to PV cells in the right ACx was not centered on the soma, especially input from L6 (asymmetry indices: *L5 to L3* = *0*.*1354* and *L6 to L3* = *0*.*2122*, n = 11 cells from 3 mice).

### Connectivity of SOM interneurons in L3 of the left and right ACx

**Figure 3:**
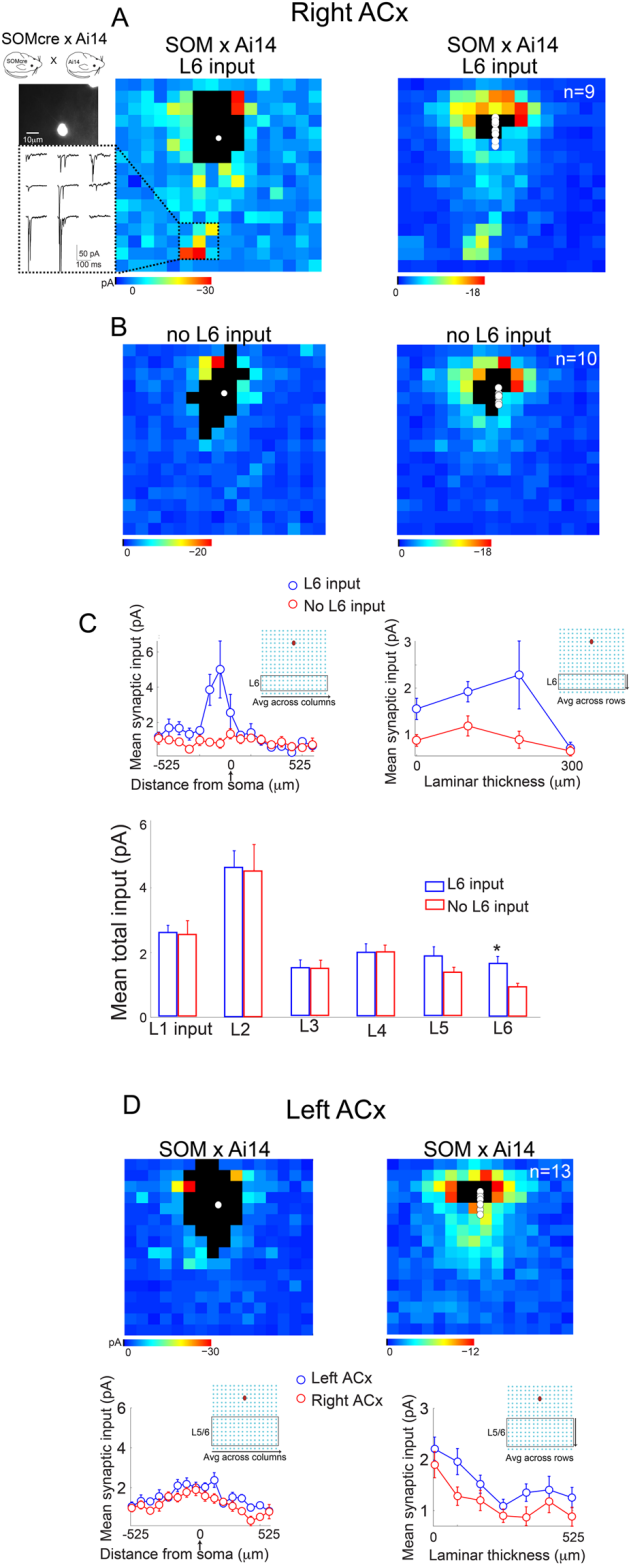
Connectivity of SOM interneurons in L3 of the ACx. (**A**) Representative single cell (left) and population (right) input maps of SOM interneurons in L3 of the right ACx that receive input from L6 (n = 9 cells from 4 mice). Panels on the left show a picture of the SOM cell mapped and representative traces of L5/6 synaptic input. (**B**) Representative single cell (left) and population (right) input maps of SOM interneurons in L3 of the right ACx that *do not* receive input from L6 (n = 10 cells from 4 mice). (**C**, top) Comparison of average synaptic input across the tonotopic axis from L6 between populations of SOM cells in the right ACx with and without input from L6. (Bottom) Comparison of total synaptic input for these two SOM populations showing that the L6 input pathway was the only significantly different source of input. (**D**) Representative single cell (left) and population (right) input maps of SOM interneurons in L3 of the left ACx (n = 13 cells from 4 mice).

Comparable to PV-positive cells, somatostatin (SOM)-expressing inhibitory interneurons are not a monolithic population; they can be further subdivided by molecular markers, firing patterns, morphology and synaptic targets^19^. We mapped the connectivity of SOM interneurons in L3 of the left and right ACx and found distinct inter- and intrahemispheric circuit-motifs. In the right ACx we found two patterns of connectivity embedded in the same layer. Approximately half of the population of SOM interneurons received input from L6 (Fig. 3A, n = 9 cells from 4 mice), whereas the other half did not (Fig. 3B, n = 10 cells from 4 mice). The difference in L6 input was significant between these two populations of SOM interneurons in the right ACx (Fig. 3C, *p* = *0*.*0093*, ranksum n = 19 cells from 4 mice). Synaptic input from other layers was not significantly different between these two populations (input from L1: *p* = *0*.*94*, L2: *p* = *0*.*98*, L3: *p* = *0*.*98*, L4: *p* = *0*.*95*, L5: *p* = *0*.*11*, ranksum n = 19 cells from 4 mice). Similar to PV cells in the right ACx, the spatial/tonotopic organization of the L6 to L3 input was not centered on the somata (Fig. 3C, top). Moreover, L6 input to SOM cells in the right ACx had the largest asymmetry of all the pathways we characterized in the study (*asymmetry index L6 to L3 SOM* = *0*.*5116*, n = 10 cells from 4 mice).

In contrast to the two distinct circuit-motifs observed in the right ACx, in the left ACx all the SOM-expressing cells mapped only received feedforward input from L4 and recurrent input from L2/3 (Fig. 3D, n = 13 cells from 4 mice). A laminar input comparison revealed no significant difference between SOM interneurons in the left ACx and those that received no L6 input in the right ACx (input from L1: *p* = *0*.*71*, L2: *p* = *0*.*97*, L3: *p* = *0*.*87*, L4: *p* = *0*.*17*, *p* = *0*.*06*, *p* = *0*.*08*, ranksum n = 23 cells from 8 mice).

**Figure 4:**
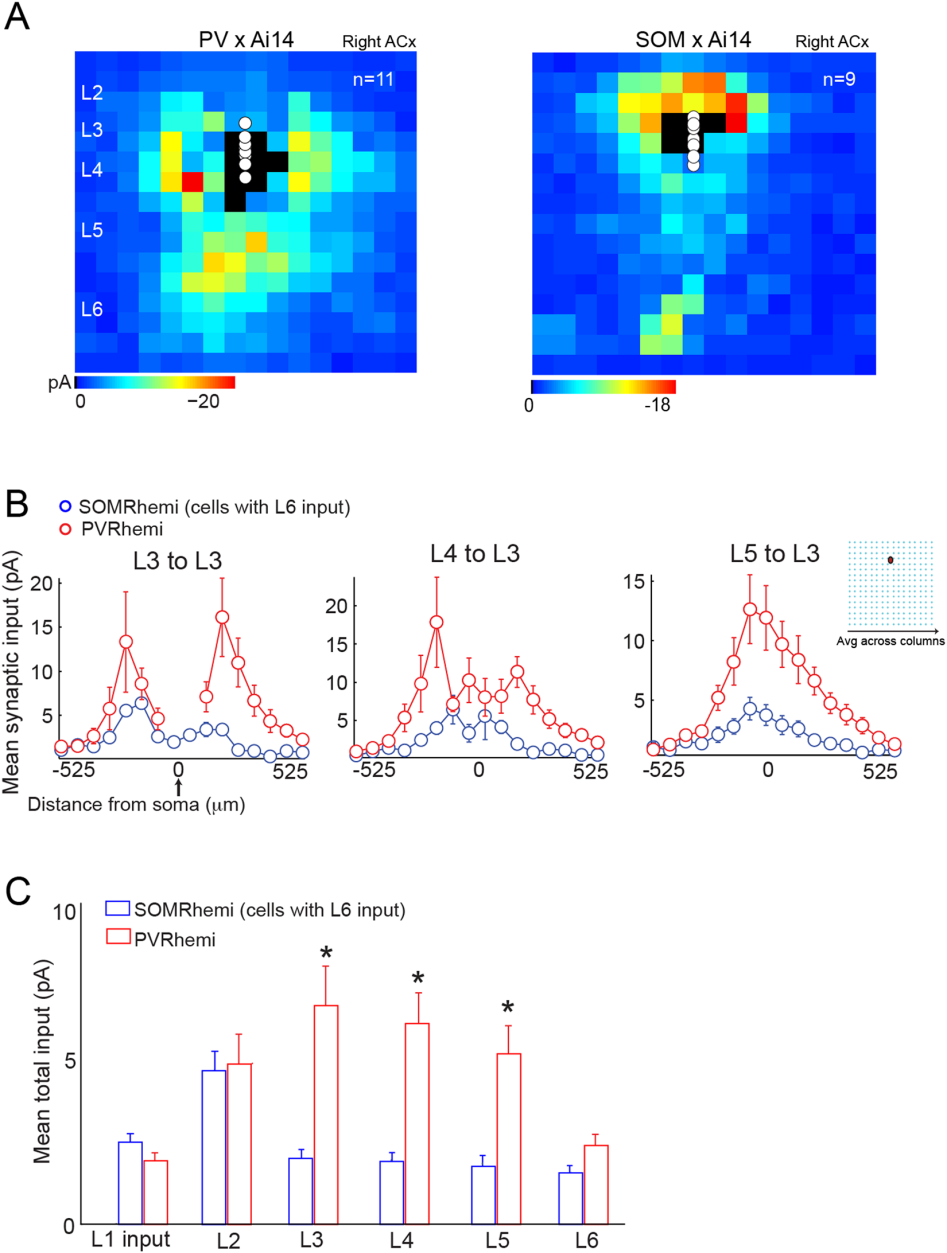
Significant differences in connectivity patterns across cell-types. (**A**) Although both PV and SOM cells in the right ACx were targeted by infragranular input, PV cells received significantly more input, particularly from L5. (**B**,**C**) In addition to input from L5, PV cells also received more feedforward input from L4 and recurrent input from L3 than SOM cells.

**Figure 5:**
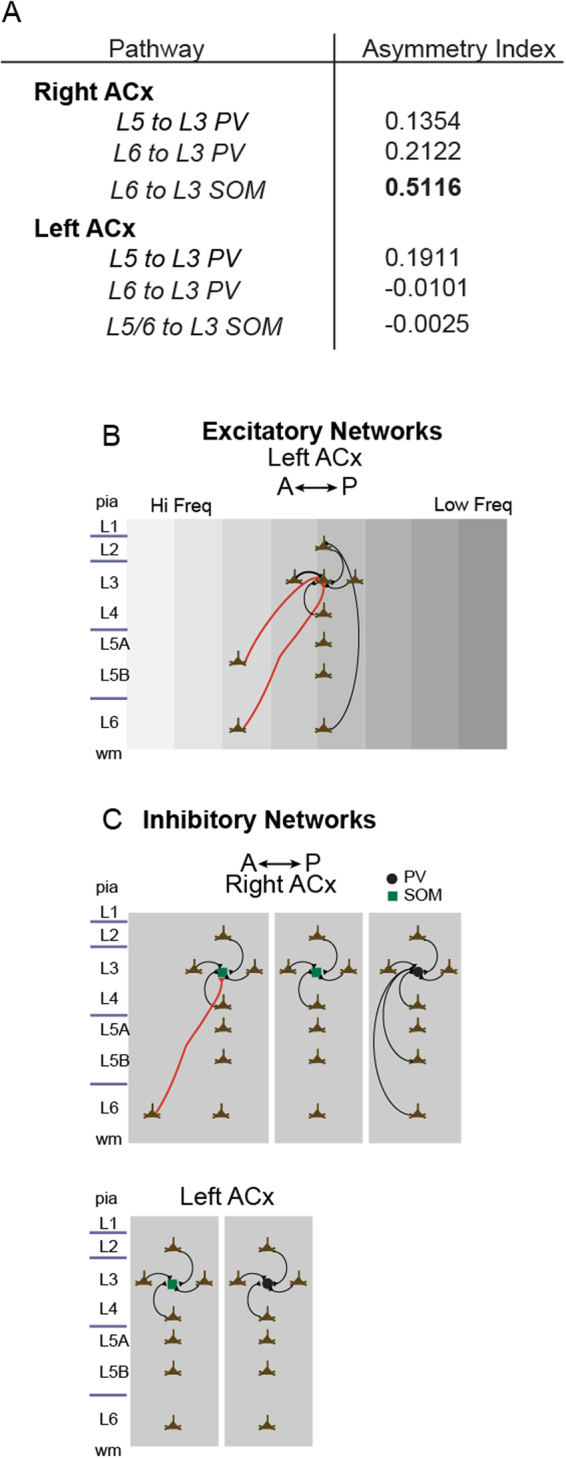
Pathway asymmetries and circuit diagram of novel and canonical circuit motifs in the ACx revealed by LSPS. (**A**) Asymmetry indices for all the infragranular pathways with significant input to SOM and PV cells. (**B**) Previously published circuit diagram of excitatory neurons in L2/3. In the left ACx, L3 is consistently sampling from intralaminar and L6 input arising from frequency areas higher than their home column. Columnar projections are labeled in black and out-of-column in red. (**C**) Circuit diagram of PV and SOM inhibitory neurons in the right (top) and left (bottom) ACx. In the left ACx, input to these inhibitory subclasses parallels what has been shown in other sensory cortices: feedforward input from L4 and recurrent connections. In contrast, in the right ACx we found novel connectivity motifs for both cell-types.

Finally, we compared synaptic input across cell classes and hemispheres (Fig. 4A). We found that PV interneurons receive more intracortical input than SOM (Fig. 4B, C). In particular, PV cells in the right ACx receive more input than SOM cells in the left and right ACx (*F* = *9*.*0510*, *p* = *9*.*6597e-005*, n = 53 cells from 18 mice, ANOVA, Bonferroni corrected for multiple comparison). The asymmetry indices of all the infragranular input pathways are summarized in Fig. 5A.

## Discussion

Previously we discovered that the asymmetric nature of frequency representation in the ACx is reflected in the connectivity of excitatory neurons in the ACx (circuit diagram in Fig. 5B). We found that this connectivity is stable from 21 to 60 days postnatal and therefore unlikely to be a transient developmental stage^1^. In the present study we uncovered distinct circuit-motifs between subpopulations of inhibitory neurons in L3 within and between the left and right ACx (Fig. 5C). Mainly, infragranular input from L5/6 to PV and a sub-population of SOM interneurons in the right ACx (but not the left ACx) emerged as a novel circuit feature. Using genetically-defined cre driver lines we have found subpopulations with different connectivity embedded in the same auditory cortical circuit. These microcircuit differences could underlie the difficulty in interpreting optogenetic manipulation based on genetically defined populations.

The molecular, morphological, and electrophysiological diversity of inhibitory neurons have been their hallmark for decades. In fact, recent efforts have focused on finding parameters to reduce the diversity of their classification. One approach is to use clustering algorithms to develop an unbiased and standard taxonomy^17^. At the same time, genetic tools have come online to target broad classes of interneurons to facilitate the study of their function^11^. In addition to their diversity, the distribution of specific subclasses varies between different areas. For example, there are more PV interneurons in primary sensory regions than elsewhere in the cortex^20^. An unresolved question has been whether specific circuit-motifs vary between cortical areas as well.

Previous connectivity studies using LSPS have revealed the largely local nature of inhibitory neural connectivity. LSPS studies in the visual and barrel cortices showed that several classes of inhibitory neurons in L2/3 receive strong feedforward input from L4 and recurrent connections within L2/3; whereas input from L5 is weak, and none arises from L6^21–23^. These results are distinctly different to the functional connectivity we report here in the right ACx using the same circuit mapping technique. Nevertheless, there is evidence of pathway-specific inhibitory microcircuits within the somatosensory cortex, where several subclasses receive differential input from lemniscal and paralemniscal pathways^12^.

The specific function of inhibitory interneurons in neural computations is still under debate^24^. This could be in part because the three cre-driver lines used with optogenetic tools target functionally heterogeneous subpopulations of interneurons^19^. Our results underscore some of the shortcomings of using these tools: within the same layer we discovered distinct connectivity patterns for the same genetically defined subclass. Although genetic tools are useful and will continue to improve, our results support that circuit-specific function motifs will be a more useful paradigm to classify inhibitory neurons^13^.

In the visual cortex, the gain of visually-driven responses has been found to be modulated via the activation of L6 pyramids that target neighboring PV interneurons with axons that arborize throughout all layers, leading to suppression of an entire cortical column^8,25^. In contrast, the L5/6 excitatory projections we discovered in the right ACx directly target interneurons in superficial layers. On the other hand, connectivity in the left ACx for both inhibitory cell types we examined is more in line with findings in the visual and barrel cortices: feedforward input from L4 and recurrent connections within L2/3 (Fig. 5C). In the ACx the role of inhibitory neurons remains poorly understood. For example, the laminar and cell-type dependent tuning properties of excitation and inhibition are unclear. Some studies report co-tuned excitation and inhibition^10^, whereas others report systematic differences across layers and cell-types^7,26,27^. Moreover, PV interneurons in L2/3 appear to be more broadly tuned than somatostatin^7^. Our results suggest that in addition to subclass coding differences, we also need to strongly consider intralaminar and hemispheric circuit-motifs in our studies and interpretation of inhibitory function in the ACx.

### Functional implications

One proposed function of the Auditory Cortex is to form neural representations of ethologically relevant, spectrotemporally complex stimuli like animal vocalizations^28^. The efficiency of decoding rapidly changing sounds is improved by hemispheric lateralization, as found for human speech^29,30^. This adaptation is also widespread in other animals, including mice, suggesting their auditory cortex may share mechanisms that can be studied using the powerful circuit-analysis tools available in this species. Functionally, the left and right ACx are postulated to specialize in processing different spectro-temporal features of sounds, but the mechanisms remain unresolved^30^. The left ACx is believed to preferentially process vocalizations^31,32^, whereas behavioral studies in rodents have shown a processing bias to detect the direction of frequency-modulated sweeps in the right ACx by using global cues^33^. Further, the right ACx of the rat has a sweep direction selectivity map in parallel with the tonotopic map^34^. In future studies we will determine whether the inhibitory connectivity motifs we found in this study play a role in sweep direction selectivity. One clue to their function may lie in the predominant tonotopic bias of the L5/6 input: most hotspots were not centered on the soma of the cell recorded (Figs 2C, 3C, 4A, 5A). Future experiments will examine if there are systematic tonotopic changes in the bias of L5/6 input in the right ACx.
